# Study on the Pressure Regulation Method of New Automatic Pressure Regulating Valve in the Electronically Controlled Pneumatic Brake Systems in Commercial Vehicles

**DOI:** 10.3390/s22124599

**Published:** 2022-06-17

**Authors:** Gangyan Li, Xiaoxu Wei, Zaiyu Wang, Hanwei Bao

**Affiliations:** School of Mechanical and Electrical Engineering, Wuhan University of Technology, Wuhan 430070, China; gangyanli@whut.edu.cn (G.L.); 259636@whut.edu.cn (X.W.); z623532_@whut.edu.cn (Z.W.)

**Keywords:** electronically controlled pneumatic brake system (ECPBS), LF automatic pressure regulating valve (LF-APRV), dynamic PWM coupling pressure regulation method, test-based analysis

## Abstract

In order to adapt the development of vehicle driving automation technology for driving conditions under different levels of automation and based on the independently invented LF automatic pressure regulating valve (LF-APRV) for electronically controlled pneumatic brake systems (ECPBS), the dynamic PWM coupling pressure regulation method is proposed. This method realizes pressure regulation by adjusting the duty cycle of the control signal of the LF-APRV at different stages in the pressure regulation cycle. A co-simulation model was established to verify the feasibility of the method, and a test system was built to verify the correctness of the co-simulation model. Through the test, the pressure regulation performance of dynamic PWM coupling pressure regulation method and conventional on/off pressure regulation method was compared. The results show that the new method can improve the stability of pressure regulation, although the response time increases; under the new method, the overshoot of the pressure rising from 0 to 0.5 MPa was reduced by 69%, and the overshoot of the pressure decreasing from 0.5 MPa to 0.2 MPa was basically 0. Finally, tests and simulations showed that the dynamic PWM coupling pressure regulation method can meet the continuous graded braking requirements of vehicles, and the pressure response has good tracking performance on the target pressure.

## 1. Introduction

The ECPBS in commercial vehicles can meet the needs for the development of driving automation. It adopts the electronically controlled braking mode and has the advantage of rapid response. The automatic pressure regulating valve (APRV) is the core component in the ECPBS of commercial vehicles, and its performance directly affects the performance of the whole braking system [1] The pressure regulation method of the APRV is the key factor affecting its pressure response characteristics. A good pressure regulation method can ensure that the APRV can meet the braking requirements of various working conditions. Therefore, study of the pressure regulation method of APRV is of great significance for the safety and comfort of commercial vehicle braking [2].

At present, the study of APRV has mainly focused on theoretical analysis and structural design. The APRV mainly relies on high-speed inlet solenoid valves and high-speed exhaust solenoid valves to complete pressure regulation. Therefore, the study of the pressure regulation methods for the APRV has mainly focused on the control method of the high-speed solenoid valve. Hanwei Bao et al. [3], based on a functional analysis of the ECPBS in commercial vehicles, put forward four different structures for the APRV. After comparing the pressure response characteristics of the four APRV structures, the structure with the best performance was determined. Wu S. [4] analyzed and compared the composition and working principle of the traditional pneumatic braking system and the ECPBS in commercial vehicles, obtaining function and performance indexes for the APRV that are suitable for ECPBS and designing an APRV composed of a one-way valve, a solenoid valve, and a relay valve. The feasibility of the design was verified by simulation and experiments, and the key structural parameters that affect the pressure regulation performance were optimized. Mujie You et al. [5] researched the proportional relay valve and verified the feasibility of the proportional relay valve as a pressure regulating element through modeling simulation and open-loop tests; they also developed a controller based on feedforward compensation for it. The test verified that the controller ccould improve the pressure regulation accuracy and robustness of the proportional relay valve. Xiaohan Li et al. [6] established the co-simulation model for the automobile’s pneumatic ABS valve by using AMESim and MATLAB/Simulink. By combining simulation and experiments, the braking performance of vehicles when the ABS valve fails and its associated patterns were studied, and the influence of different fault forms of the ABS valve on vehicle braking was obtained through comparison. Long X. [7] proposed a new structural scheme for a proportional relay valve, established its mathematical model and AMESim simulation model, and obtained the optimal combination of the key structural parameters for the proportional relay valve through orthogonal experiments, also designing a control algorithm based on Fuzzy PID and PWM (pulse width modulation) control for the proportional relay valve. Dawei Hu et al. [8] established a nonlinear model for the pneumatic braking system, and on this basis, the linear variable parameter (LPV) model of the braking system was established. Additionally, a model predictive control (MPC) controller with a Kalman filter was designed for the LPV model. The experiment verified that the controller had good control and tracking performance. Aladjev V et al. [9] analyzed and summarized the reasons affecting the braking effect of vehicles. Considering the principle and the elements of the pneumatic brake system, mathematical models for the pneumatic brake system were presented. The hyperbolic equation was used to describe the flow state of the gas in the pipeline of the pneumatic brake system, and the characteristics method was used to solve the equation. Diao Yong. [10] established a mathematical model and simulation model for the pneumatic solenoid valve and built a response characteristic test system for a solenoid valve composed of a pneumatic circuit module, a signal acquisition module, and a control module. Through experiments, measurement problems related to parameters such as response time and the starting pressure of the pneumatic solenoid valve were solved. Tristan Braun et al. [11] established a mathematical model of distributed parameters for a cylindrical double-coil solenoid valve, developed a solenoid valve control strategy based on feedforward control with the help of the mathematical model, and verified the feasibility of the control strategy through experiments. Bin Zhang et al. [12] developed a self-correcting PWM control algorithm for a hydraulic high-speed on/off valve. Through experiments, it was verified that this control algorithm was able to improve the opening speed and dynamic response characteristics of the high-speed on/off valve. Peng Liu et al. [13] optimized the dynamic response speed of a high-speed solenoid valve based on the response surface method and genetic algorithm, which provided theoretical guidance for the design and performance optimization of the high-speed solenoid valve. Lisha Zhang et al. [14] designed a feedforward fuzzy PID controller by using LabVIEW software. The pressure response characteristics of APRV under PID control, feedforward PID control, and feedforward fuzzy PID control were obtained through experiments. The comparison of the test results shows that feedforward fuzzy PID control can reduce response time and overshoot. Rui Zhang et al. [15] established a dynamic model of pneumatic braking system by using the hybrid logic dynamic representation method. A hybrid MPC method was proposed for implementing the multi-objective optimization. The test and simulation results showed that this method could directly and effectively reduce the switching frequency of the valve and ensure the accurate control of pressure.

At present, most research is aimed at the existing pressure regulating components of pneumatic braking systems, such as proportional relay valves, ABS valves, and others, by proposing effective pressure regulating methods or strategies to control the pressure regulating components. Research on solenoid valves is mainly focused on a single-solenoid valve, and the precise control of the output pressure or flow of the solenoid valve is achieved by implementing control strategies. There is little research, however, on the coordinated control of multiple solenoid valves. Based on the independently invented LF-APRV in commercial vehicle ECPBS for driving automation [16], and according to the characteristic that the LF-APRV requires multiple solenoid valves to work together in order to complete pressure regulation, this paper proposes the dynamic PWM coupling pressure regulation method for a new APRV. Based on the detailed analysis of its working principle, the co-simulation model of the LF-APRV and its pressure regulation method were established by using AMESim software and MATLAB/Simulink software to verify the feasibility of the dynamic PWM coupling pressure regulation method. The performance test system for the pressure regulation method was built, and the correctness of the simulation model was verified by testing. Through comparisons with the on/off pressure regulation method, the advantages of the dynamic PWM coupling pressure regulation method were verified. Finally, the pressure response characteristics of the dynamic PWM coupling pressure regulation method under various complex braking conditions were obtained through simulation and testing, and its pressure regulation performance was evaluated.

## 2. Working Principle of the ECPBS and the LF-APRV for Driving Automation in Commercial Vehicles 

### 2.1. Working Principle of the ECPBS for Driving Automation in Commercial Vehicles

**Figure 1:**
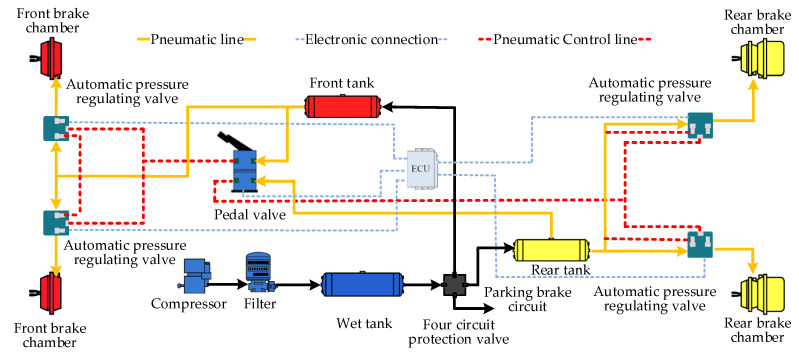
Principle drawing of the ECPBS for driving automation in commercial vehicles.

Driving automation is an inevitable trend in vehicle development. The autonomous driving system includes the perception layer, the decision-making layer, and the execution layer, and the ECPBS is one of the important components of the execution layer of the autonomous driving system. The ECPBS used in commercial vehicles can well meet the needs of driving automation. It adds an electronic control function [17] so that it can cooperate with the autonomous driving system through the interaction of electronic signals. The perception layer of the autonomous driving system senses the external environment and transmits the environmental information to the decision-making layer. After processing the information, the decision-making layer generates a control signal for the ECPBS and realizes the real-time monitoring and control of the braking process [18]. Figure 1 is the principle drawing of the ECPBS for driving automation in commercial vehicles [3]. It can be seen from the figure that the APRV is installed in the front of each wheel brake chamber. The APRV is the core of the commercial vehicle ECPBS in commercial and can realize the independent adjustment of the pressure of each brake chamber.

### 2.2. Working Principle of the LF-APRV in the ECPBS of Commercial Vehicles for Driving Automation

**Figure 2:**
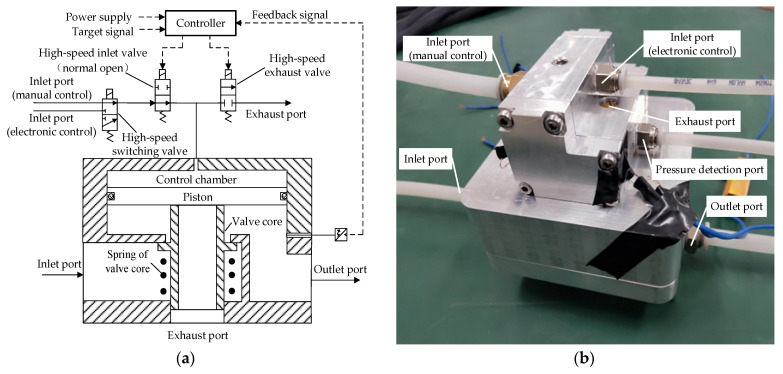
The LF-APRV in the ECPBS for driving automation of commercial vehicles: (**a**) principle drawing and (**b**) photograph.

The APRV is the key component that distinguishes the ECPBS from the traditional pneumatic braking system. The APRV must be able to complete electronically controlled braking. In addition, it must also be able to complete braking when the electronic control fails. Figure 2 shows the LF-APRV, which is composed of high-speed switching valve, high-speed inlet valve, high-speed exhaust valve, and relay valve [3]. The high-speed switching valve can realize the switching of two gas circuits. The high-speed inlet valve and high-speed exhaust valve can only be opened or closed to control the on/off status of the gas circuit. The three high-speed solenoid valves coordinate to regulate the pressure in the control chamber of the relay valve. When the pressure in the control chamber increases, the piston and valve core move downward under the action of compressed gas and open the inlet valve. The compressed gas at the inlet port can control the brake chamber through the inlet valve to complete braking; when the pressure in the control chamber decreases, the piston and valve core move upward and close the inlet valve. When the pressure in the control chamber continues to decrease, the piston continues to move upward, but the valve core remains stationary and the piston and valve core separate so the exhaust valve opens, and the gas at the outlet port is discharged into the atmosphere, reducing the pressure in the brake chamber and releasing the brake.

The LF-APRV can control the relay valve through the cooperative work of three high-speed solenoid valves to complete electronic control braking, manual control braking, and emergency braking in the case of electric control failure. The high-speed switching valve is mainly responsible for the switching between the electronic control circuit and the manual control circuit. The high-speed switching valve is powered on to connect the electronic control circuit. The high-speed inlet valve and the high-speed exhaust valve adjust the output pressure of the LF-APRV through continuous action. When the system is in the manually controlled braking mode or when the electronic control fails, the three high-speed solenoid valves return to the default state at the same time. The high-speed switching valve connects to the manual control circuit, the high-speed inlet valve is open as normal, and the high-speed exhaust valve is closed as normal. The compressed air from the pedal valve can control the relay valve through the high-speed switching valve and the high-speed inlet valve to complete the braking.

## 3. Theoretical Analysis and Simulation Model of Pressure Regulation Characteristics of the LF-APRV in the ECPBS in Commercial Vehicles

### 3.1. Theoretical Analysis of Pressure Regulation Characteristics of the LF-APRV in the ECPBS in Commercial Vehicles

The LF-APRV is mainly composed of a high-speed solenoid valve and a relay valve. Their flow characteristics and the movement of the piston and valve core jointly determine the outlet pressure response of the LF-APRV. The solenoid valve and relay valve open or close through the movement of the valve core, and the movement of the valve core changes the flow area of the valve port. Therefore, the valve port is equivalent to a variable orifice, and the mass flow rate of the gas flowing through the variable orifice can be calculated by Equation (1) [19]:

(1) $$G=\{\begin{array}{cc} SP_{u}\cdot \sqrt{\frac{\lambda }{RT}}{\left(\frac{2}{\lambda +1}\right)}^{\frac{\lambda +1}{2(\lambda -1)}} & 0<\frac{P_{b}}{P_{u}}\leq b\\ SP_{u}\cdot \sqrt{\left[{\left(\frac{P_{b}}{P_{u}}\right)}^{\frac{2}{\lambda }}-{\left(\frac{P_{b}}{P_{u}}\right)}^{\frac{\lambda +1}{\lambda }}\right]\frac{2\lambda }{RT(\lambda -1)}} & b<\frac{P_{b}}{P_{u}}<1 \end{array}$$

where *G* is the gas mass flow rate (kg/s), *T* is the absolute temperature of gas (K), *P_u_* is upstream pressure (Pa), *R* is the gas constant, *P_b_* is the downstream pressure (Pa), *b* is the critical pressure ratio, *S* is the effective circulation area (m^2^), and λ is the thermal insulation coefficient.

The volume of the control chamber and the outlet chamber of the LF-APRV change with the movement of the valve core, so both are variable volume chambers. The inflation and deflation time of the chambers in LF-APRV is very short, so the whole process can be regarded as an adiabatic process [20]. Therefore, the pressure change in each chamber of the LF-APRV can be calculated according to Equation (2):

(2) $$\frac{dp}{dt}=\lambda \left(\frac{GRT}{V_{0}+\Delta V}-\frac{P}{V_{0}+\Delta V}\frac{d(V_{0}+\Delta V)}{dt}\right)$$

where *p* is the pressure of the chamber (Pa), *V*_0_ is the volume of the chamber (m^3^), and ΔV is the change of chamber volume when valve core moves (m^3^).

In the process of pressurization, the motion state of the piston and valve core of the LF-APRV is consistent, so they can be considered as a whole. The forces acting on them are the gas pressure on the upper and lower surfaces of the piston, friction force, viscous damping force and spring force. The motion equation of the valve core in the process of pressurization is as follows:

(3) $$\left(m_{1}+m_{2})\frac{d^{2}x}{dt^{2}}=(m_{1}+m_{2})g+P_{1}A_{1}-P_{2}A_{2}-\mathrm{sgn}(\frac{dx}{dt})\cdot c_{1}\frac{dx}{dt}-\mathrm{sgn}(\frac{dx}{dt})\cdot F_{f}-k(x+x_{0}\right)$$

In the process of decompression, the valve core is separated from the piston, and the piston keeps moving continuously. The forces on the piston are the gas pressure on the upper and lower surfaces of the piston, friction force, and viscous damping force. The piston motion equation [3] is as follows:

(4) $$m_{1}\frac{d^{2}x}{dt^{2}}=m_{1}g+P_{1}A_{1}-P_{2}A_{2}-\mathrm{sgn}(\frac{dx}{dt})\cdot c_{2}\frac{dx}{dt}-\mathrm{sgn}(\frac{dx}{dt})\cdot F_{f}$$

where *m*_1_ is the mass of piston (kg), *m*_2_ is the mass of valve core (kg), *x*_0_ is initial deflection of the spring of valve core (N), *x* is the displacement of the piston (m), *A*_1_ is the upper surface area of the piston (m^2^), *P*_2_ is the outlet pressure (Pa), *c*_1_ is the viscous damping coefficient of the valve core (N/(m/s)), *P*_1_ is the pressure of the control chamber (Pa), *c*_2_ is the viscous damping coefficient of the piston (N/(m/s)), *A*_2_ is the lower surface area of the piston (m^2^), *k* is the spring stiffness of the valve core (N/m), and *F_f_* is the friction force on the piston (N).

### 3.2. Simulation Model of Pressure Regulation Characteristics of the LF-APRV in the ECPBS in Commercial Vehicles

**Figure 3:**
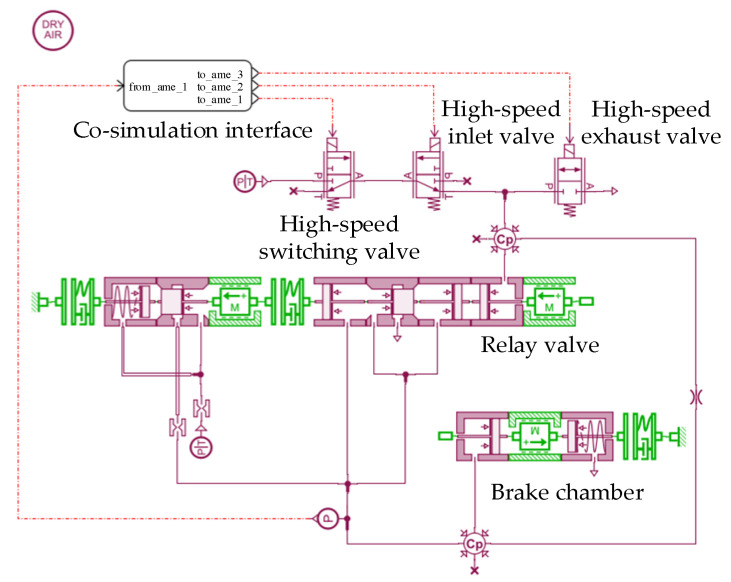
The AMESim model of pressure regulation characteristics of the LF-APRV.

In order to study the pressure regulation characteristics of the LF-APRV according to the valve port flow characteristic equations and valve core motion equations, the pressure regulation characteristic simulation model of the LF-APRV, as shown in Figure 3, was built by using AMESim software. The simulation model for the pressure regulation characteristics includes a high-speed switching valve model, high-speed inlet valve model, high-speed exhaust valve model, relay valve model and brake chamber model. The high-speed switching valve receives the control signal and switches between the electronically controlled braking mode and the manually controlled braking mode. The high-speed inlet valve and the high-speed exhaust valve receive the control signal and control the relay valve to complete the pressure regulation.

## 4. Dynamic PWM Coupling Pressure Regulation Method of the LF-APRV and the Simulation Model

### 4.1. Dynamic PWM Coupling Pressure Regulation Method of the LF-APRV

The LF-APRV achieves the purpose of accurate pressure regulation by accurately controlling the high-speed inlet valve and the high-speed exhaust valve. The common pressure regulation methods are the PWM pressure regulation method and the on/off pressure regulation method. The PWM pressure regulation method uses PWM signals with different duty cycles to control the action of the high-speed inlet valve and the high-speed exhaust valve and to realize the regulation of the outlet pressure of the APRV [21]. PWM pressure regulation is relatively stable, but the pressure response is slow [22]. When the duty cycle of the control signal is 0 or 1, the high-speed inlet valve and the high-speed exhaust valve only have two states, open and closed, which is called the on/off pressure regulation method. On/off pressure regulation has fast pressure response, but the pressure regulation accuracy is poor [23]. In this paper, the dynamic PWM coupling pressure regulation method is proposed to control the high-speed inlet valve and the high-speed exhaust valve to realize the accurate regulation of braking pressure.

The LF-APRV must be able to complete pressurization, pressure maintenance, and pressure reduction during the working process in order to realize the continuous braking and release of the vehicles. To ensure fast and stable pressure regulation, the working modes of the LF-APRV are divided into fast pressurization mode, slow pressurization mode, pressure-maintaining mode, fast decompression mode, and slow decompression mode. The dynamic PWM coupling pressure regulation method can adjust the duty cycle of the control signals in different working modes to switch between the PWM pressure regulation and on/off pressure regulation methods. The details of each mode are as follows.

(1) Fast pressurization working mode: At the beginning of the pressurization process, the outlet pressure of the LF-APRV needs to quickly approach the target pressure, so the on/off pressure regulation method is adopted. The high-speed inlet valve is open and the high-speed exhaust valve is closed, and the duty cycles of the control signals of the two high-speed solenoid valves are both 0.
(2) Slow pressurization working mode: When the outlet pressure of the LF-APRV is close to the target pressure. The PWM pressure regulation method is adopted to reduce the overshoot and ensure the stability of the pressure regulation, and the control signals of the high-speed inlet valve and high-speed exhaust valve are set respectively as PWM signals with duty cycles of *C*_1_ and *C*_2_.
(3) Pressure-maintaining working mode: When the outlet pressure of the LF-APRV is equal to the target pressure or the difference is within a certain range, the high-speed inlet valve and high-speed exhaust valve close, and the duty cycle of the control signal of the high-speed inlet valve is 1 and the duty cycle of the control signal of the high-speed exhaust valve is 0.
(4) Fast decompression working mode: At the beginning of the decompression process, the outlet pressure of the LF-APRV needs to be released quickly, so the on/off pressure regulation method is adopted to realize rapid decompression, and the duty cycle of the control signals of the two high-speed solenoid valves are both 1.
(5) Slow decompression working mode: To reduce the overshoot and maintain the stability in the decompression process, the PWM pressure regulation method is adopted. The control signals the of the high-speed inlet valve and high-speed exhaust valve are respectively set as PWM signals with duty cycles of *C*_3_ and *C*_4_ to slow the pressure reduction.

The definition of the five working modes of the LF-APRV is realized by the deceleration threshold T*_d_* and the pressure-holding threshold T*_h_*. The model for the dynamic PWM coupling pressure regulation method is expressed in Equation (5):

(5) $$(K_{1},K_{2})=\left\{ \begin{array}{cc} K_{1}=0,K_{2}=0 & P_{t}-P_{d}>T_{d}\\ K_{1}=C_{1},K_{2}=C_{2} & T_{h}<P_{t}-P_{d}\leq T_{d}\\ K_{1}=1,K_{2}=0 & -T_{h}\leq P_{t}-P_{d}\leq T_{h}\\ K_{1}=1,K_{2}=1 & P_{t}-P_{d}<-T_{d}\\ K_{1}=C_{3},K_{2}=C_{4} & -T_{d}\leq P_{t}-P_{d}<-T_{h} \end{array}\right.$$

where *K*_1_ and *K*_2_ are the duty cycles of the control signals of the high-speed inlet valve and the high-speed exhaust valve, *P_t_* is the target pressure of the LF-APRV (Pa), *P_d_* is the output pressure of the LF-APRV (Pa), T*_d_* is the deceleration threshold (Pa), and T*_h_* is the pressure holding threshold (Pa).

### 4.2. Simulation Model of the Dynamic PWM Coupling Pressure Regulation Method of the LF-APRV

**Figure 4:**
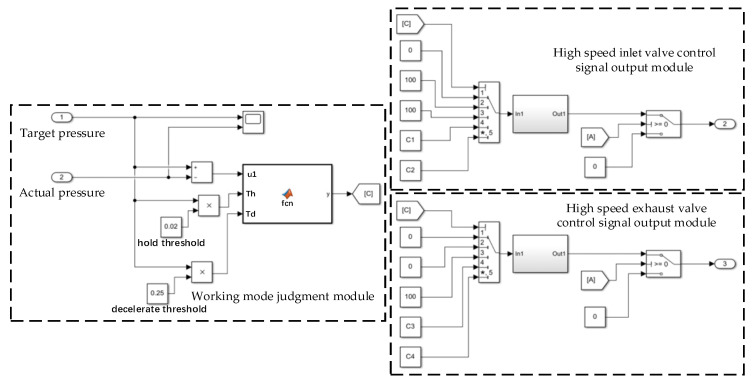
The MATLAB/Simulink model of dynamic PWM coupling pressure regulation method.

The simulation model of the dynamic PWM coupling pressure regulation method established by MATLAB/Simulink includes a working mode judgment module and control signal output modules for the high-speed inlet valve and high-speed exhaust valve; Figure 4 depicts the simulation model. The input of the working mode judgment module is the target pressure signal and the outlet pressure feedback signal of the LF-APRV. It calculates the difference between the two input signals and compares the difference with the deceleration threshold Td and pressure-holding threshold Th, and then it outputs the working mode number according to the comparison results. The high-speed inlet valve control signal output module and the high-speed exhaust valve control signal output module output the PWM signal or the on/off signal according to the working mode number in order to signal the LF-APRV to switch between the different working modes.

### 4.3. Co-Simulation Model for the LF-APRV and the Dynamic PWM Coupling Pressure Regulation Method

**Figure 5:**
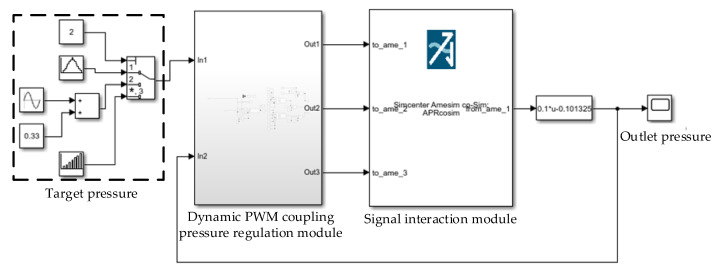
The control part of the co-simulation model.

To verify the feasibility of the dynamic PWM coupling pressure regulation method, a co-simulation model was established by using the simulation model of the dynamic PWM coupling pressure regulation method and the established AMESim model of pressure regulation characteristics of the LF-APRV. The co-simulation model is composed of a control part and a pneumatic circuit part. The control part is shown in Figure 5 and the pneumatic circuit part is shown in Figure 3. The control part includes a target pressure module, a dynamic PWM coupling pressure regulation module, and a signal-interaction module. The signal-interaction module creates the signal interaction between MATLAB/Simulink model and the AMESim model. The dynamic PWM coupling pressure regulation module receives the outlet pressure signal of the LF-APRV from the AMESim model and compares it with the target pressure signal, and then it outputs the control signals from high-speed inlet valve and high-speed exhaust valve.

According to research on the LF-APRV in the ECPBS in commercial vehicles, the parameters of the co-simulation model are shown in Table 1.

**Figure 6:**
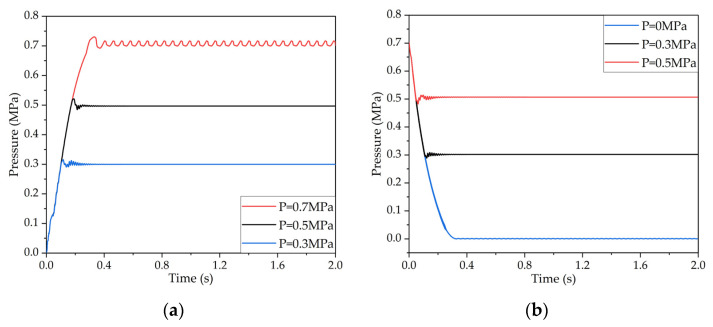
Simulation curves of the pressure regulation characteristics during pressurization and decompression of the LF-APRV: (**a**) pressurization response curve and (**b**) decompression response curve.

The air source pressure of the co-simulation model was set at 0.75 Mpa, the target pressure was set as 0.7 Mpa, 0.5 Mpa and 0.3 Mpa in the pressurization process, and the target pressure was set at 0.5 Mpa, 0.3 Mpa, and 0 Mpa in the decompression process. The pressurization and decompression response curves of the LF-APRV were obtained through simulation, and are shown in Figure 6. The results show that the pressure response had overshoot; because the dynamic PWM coupling pressure regulation needs to continuously adjust the outlet pressure according to the difference between the outlet pressure and the target pressure, and the whole pressure regulation is a dynamic and uninterrupted process. Therefore, the actual outlet pressure may not be stable and may fluctuate around the target pressure. In general, however, the dynamic PWM coupling pressure regulation method could control the LF-APRV to complete pressurization and decompression, which verifies the feasibility of the dynamic PWM coupling pressure regulation method.

## 5. Test and Analysis of the Pressure Regulation Method of the LF-APRV in ECPBS in Commercial Vehicles

### 5.1. Performance Test System of Dynamic PWM Coupling Pressure Regulation Method of the LF-APRV

**Figure 7:**
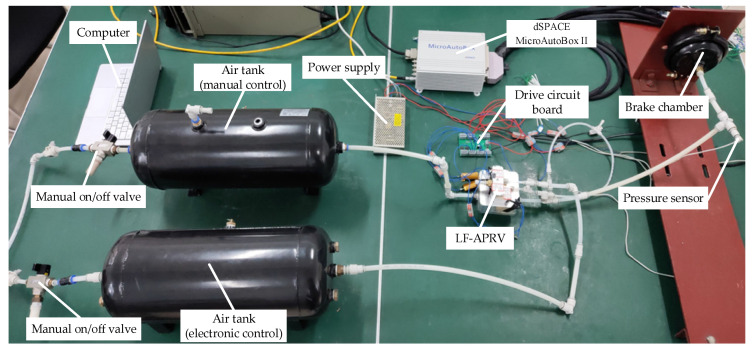
Performance test system of dynamic PWM coupling pressure regulation method of the LF-APRV.

To test and evaluate the pressure regulation performance of the dynamic PWM coupling pressure regulation method of the LF-APRV, a test system for the pressure regulation method of the LF-APRV was built. As shown in Figure 7, the test system includes a manual on/off valve, air tanks, an LF-APRV, a brake chamber, a dSPACE MicroAutoBox II real-time system, a power supply, a drive circuit board, a pressure sensor, etc. The air tanks include an electronic control air tank and a manual control air tank, which are responsible for providing compressed gas to the electronic control circuit and the manual control circuit, respectively. The pressure regulation method program was written by using MATLAB/Simulink, and was downloaded into dSPACE MicroAutoBox II. dSPACE MicroAutoBox II ran the pressure regulation method program and output the control signals for the high-speed switching valve, the high-speed inlet valve, and the high-speed exhaust valve. The drive circuit board could amplify the signals output by dSPACE MicroAutoBox II from 5 V to 24 V and drive the high-speed solenoid valves. The pressure regulation method program receives the outlet pressure feedback signal from the pressure sensor and compares the outlet pressure signal with the target pressure signal, and then it switches between the different working modes according to the difference between the two and quickly and accurately adjusts the outlet pressure of the LF-APRV.

### 5.2. Verification of the Co-Simulation Model for the LF-APRV and the Dynamic PWM Coupling Pressure Regulation Method

**Figure 8:**
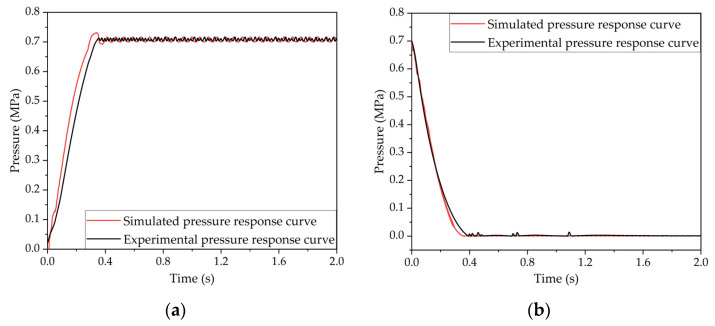
Verification of the co-simulation model for the LF-APRV and the dynamic PWM coupling pressure regulation method: (**a**) pressurization response curves and (**b**) decompression response curves.

The air source pressure of the co-simulation model and the test system was set at 0.75 MPa, and the target pressure of the LF-APRV was set at 0.7 MPa. In the slow pressurization mode, the duty cycle of the control signal of the high-speed inlet valve was 0.4 (*C*_1_ = 0.4), and the duty cycle of the control signal of the high-speed exhaust valve was 0 (*C*_2_ = 0). In the slow decompression mode, the duty cycle of the control signal of the high-speed inlet valve was 1 (*C*_3_ = 1), and the duty cycle of the control signal of the high-speed exhaust valve was 1 (*C*_4_ = 1). The pressurization and decompression response curves of the LF-APRV were obtained through simulation and test and are shown in Figure 8.

The simulation results show that the time when the outlet pressure of the LF-APRV reached 75% of the target pressure was 0.18 s, and the time when the pressure decreased from 0.7 MPa to 0 was 0.35 s. The test results show that the time when the outlet pressure of the LF-APRV reached 75% of the target pressure was 0.23 s, and the time when the pressure decreased from 0.7 MPa to 0 was 0.39 s. Due to the simplification of the simulation model, the simulated pressure response time was shorter, and the overshoot of the simulation pressure response was more serious during the pressurization process. Generally speaking, the simulation results are very close to the test results and have good consistency.

## 6. Test-Based Pressure Regulation Performance Analysis of the Dynamic PWM Coupling Pressure Regulation Method of the LF-APRV

### 6.1. Comparative Analysis of Pressure Regulation Performance of Different Pressure Regulation Methods

**Figure 9:**
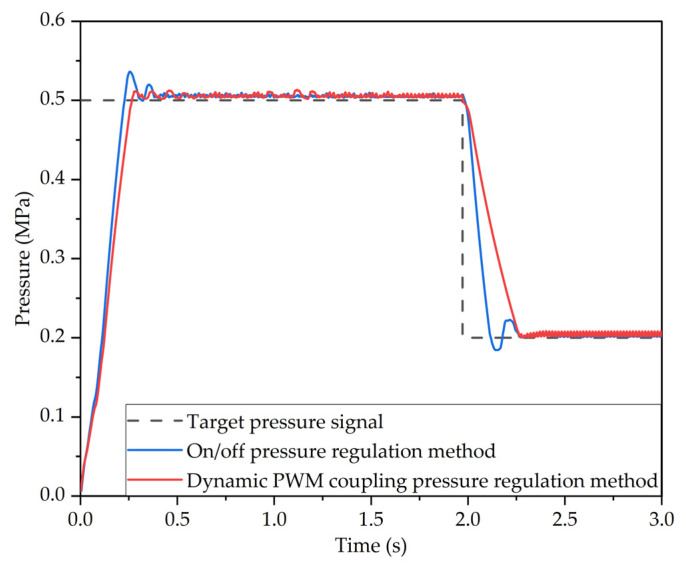
The outlet pressure response characteristic curve of the LF-APRV with different pressure regulation modes.

The control variable tests show that the dynamic PWM coupling pressure regulation method has good pressure regulation performance when the parameters of the slow pressurization mode and slow decompression mode are *C*_1_ = 0.7, *C*_2_ = 0.5, *C*_3_ = 0.7 and *C*_4_ = 1. The target pressure of the LF-APRV was set at 0.5 MPa and then decreased to 0.2 MPa after 2 s. The outlet pressure response characteristic curve of the LF-APRV under the control of the dynamic PWM coupling pressure regulation method was obtained when *C*_1_ = 0.7, *C*_2_ = 0.5, *C*_3_ = 0.7, and *C*_4_ = 1. The outlet pressure response characteristic curve of the LF-APRV under on/off pressure regulation method was obtained when *C*_1_ = 0, *C*_2_ = 0, *C*_3_ = 1, and *C*_4_ = 1. Figure 9 shows the outlet pressure response characteristic curve of the LF-APRV under different pressure regulation methods.

It can be seen from Figure 9 that, under the on/off pressure regulation method, it takes 0.17 s for the outlet pressure of the LF-APRV to reach 75% of the target pressure and 0.13 s for the pressure to decreases from 0.5 MPa to 0.2 MPa, but it takes 0.27 s for the pressure to stabilize; the overshoot during the pressurization and decompression processes was 7.2% and 8%, respectively. Under the dynamic PWM coupling pressure regulation method, it takes 0.20 s for the outlet pressure of the LF-APRV to reach 75% of the target pressure, and 0.28 s for the pressure to decreases from 0.5 MPa to 0.2 MPa; the overshoot during the pressurization process was 2.2%, and there was no overshoot during the decompression process.

Compared with the on/off pressure regulation method, the dynamic PWM coupling pressure regulation method increases the response time of the outlet pressure of the LF-APRV, but the pressure response time still meets the requirements. The overshoot of the outlet pressure response is significantly reduced, which improves the accuracy and stability of the pressure regulation.

### 6.2. Pressure Regulation Performance Analysis of the Dynamic PWM Coupling Pressure Regulation Method under Complex Braking Conditions

**Figure 10:**
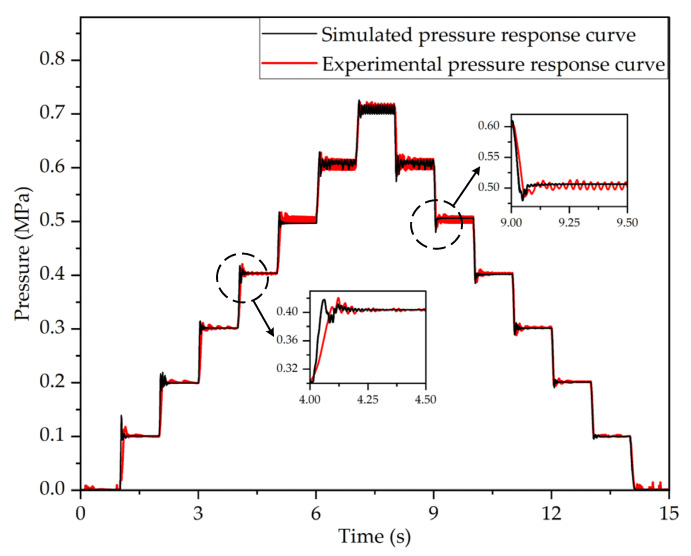
The outlet pressure response characteristics curves of the LF-APRV under continuous graded braking conditions obtained by test and simulation.

To verify the pressure regulation performance of the dynamic PWM coupling pressure regulation method under complex braking conditions and to comprehensively evaluate its pressure regulation performance, the outlet pressure response characteristics curves of the LF-APRV under several complex target pressures were obtained through testing and simulation. The target pressure signal was set as a continuous step pressure signal to simulate the working state of the LF-APRV under the conditions of continuous graded braking. Figure 10 shows the outlet pressure response characteristics curve of the LF-APRV under the continuous graded braking conditions obtained by test and simulation.

Figure 10 shows that the outlet pressure response characteristics curves obtained from the test and simulation were basically consistent. When the target pressure is low, the outlet pressure response has bigger overshoot, but it can quickly reach the target pressure. When the target pressure increases, the outlet pressure response quickly reaches the target pressure but eventually fluctuates around the target pressure. The error in the fluctuation process is less than ±2%, which meets the requirements of the standard ISO 6953-2:2000. Therefore, the PWM coupling pressure regulation method can meet the requirements for continuous graded braking.

**Figure 11:**
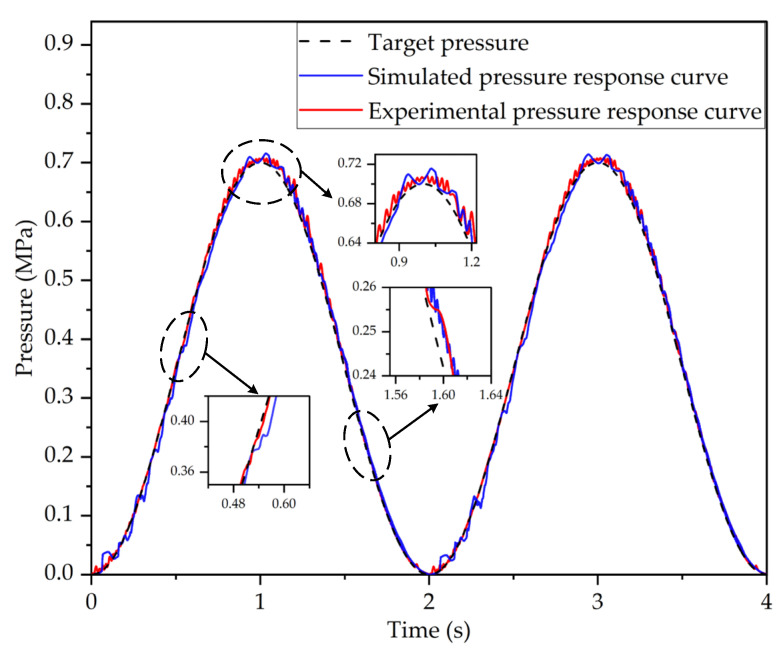
The outlet pressure response characteristics curves of the LF-APRV under sinusoidal target pressure obtained by test and simulation.

The target pressure signal was set as a sinusoidal signal with a frequency of 0.5 Hz, an amplitude of 0.35 MPa, and an offset of 0.35 MPa. Figure 11 shows the pressure response characteristics curves of the LF-APRV under sinusoidal target pressure. It can be seen from Figure 11 that, in a sinusoidal signal cycle, the outlet pressure response of the LF-APRV fluctuates in the initial and middle stages of pressure regulation, and the simulated curve fluctuates more seriously. However, on the whole, the pressure response curve obtained from the testing and simulation can maintain good consistency with the target pressure, and the dynamic PWM coupling pressure regulation can follow the target pressure signal well.

## 7. Conclusions

Based on the independently invented LF-APRV in the ECPBS of commercial vehicles, a dynamic PWM coupling pressure regulation method is proposed. The mathematical models of the LF-APRV and the dynamic PWM coupling pressure regulation method were established, andnd the co-simulation model was established by using AMESim software and MATLAB/Simulink software to verify the feasibility of the pressure regulation method. The performance testing system of the LF-APRV was built to verify the correctness of the simulation model. Through the tests, it is concluded that the dynamic PWM coupling pressure regulation method has good pressure regulation effects when the parameters of the slow pressurization mode and the slow decompression mode are *C*_1_ = 0.7, *C*_2_ = 0.5, *C*_3_ = 0.7, and *C*_4_ = 1. Based on the tests, the pressure regulation performance of the dynamic PWM coupling pressure regulation method of the LF-APRV was analyzed. Compared with the on/off control pressure regulation method, the new method can improve the stability of pressure regulation, but the response time increases; the overshoot of pressure rising from 0 to 0.5 MPa under the control of the dynamic PWM coupling pressure regulation method was reduced by 69%, and the overshoot of pressure decreasing from 0.5 MPa to 0.2 MPa was 0, and the response time meets the requirements. The dynamic PWM coupling pressure regulation method can also complete the pressure regulation under complex braking conditions, and the outlet pressure response can follow the target pressure signal well.

In future research, we will apply the LF-APRV to commercial vehicles, design control strategies for multiple LF-APRV to work together, and conduct more in-depth research in combination with the overall structure and driving conditions of commercial vehicles, making contributions to the safety and stability of commercial vehicle braking and the development of automatic driving technology.

## Figures and Tables

**Table 1:** Parameters of the co-simulation model for the LF-APRV and dynamic PWM coupling pressure regulation method.

Parameter	Air Source Pressure	Driving Voltage	Frequency of Solenoid Valve	Orifice Area of Solenoid Valve	Volume of Control Cavity
Values	0.75 MPa	24 V	80 Hz	2.5 mm^2^	4 × 10^−5^ m^3^
Parameter	Volume of brake chamber	Mass of the valve core	Maximum travel	Pre-load force	Spring rate
Values	3 × 10^−4^ m^3^	5 g	0.24 mm	10 N	2000 N/m

## Data Availability

The study did not report any data.
